# The effectiveness of VIPP-V parenting training for parents of young children with a visual or visual-and-intellectual disability: study protocol of a multicenter randomized controlled trial

**DOI:** 10.1186/s13063-015-0916-6

**Published:** 2015-09-09

**Authors:** Mathilde M. Overbeek, Paula S. Sterkenburg, Sabina Kef, Carlo Schuengel

**Affiliations:** Department of Clinical Child and Family Studies, VU University, Van der Boechorststraat 1, 1081 BT Amsterdam, The Netherlands; EMGO+ Institute for Health and Care Research, VU University Medical Center, Amsterdam, The Netherlands; Bartiméus, Doorn, The Netherlands

**Keywords:** Visual impairment, Visual-and-intellectual disability, Early intervention, Video-feedback, Sensitive responsiveness, Parent-child interaction, Parenting, Attachment, Parental self-efficacy, Parenting stress

## Abstract

**Background:**

Visual or visual-and-intellectual disabilities of children make daily interactions more difficult for their parents and may impact the quality of the parent-child relationship. To support these parents, an existing intervention (Video-feedback Intervention to promote Positive Parenting; VIPP; Juffer F, Bakermans-Kranenburg MJ, van IJzendoorn MH, 2008. Promoting positive parenting; an attachment-based intervention. Mahwah, NJ: Lawrence Erlbaum Associates; 2008) was adapted for use with parents of children with a visual or visual-and-intellectual disability (VIPP-V). This attachment-based intervention was hypothesized to support parents’ interpretation and understanding of the behavior of their child with a visual or visual-and-intellectual disability and respond to their child’s signals in a sensitive way to improve parent-child interaction quality.

**Methods/Design:**

A randomized controlled trial (RCT) will be conducted to assess the effectiveness of the adapted intervention VIPP-V (Video-feedback Intervention to promote Positive Parenting in parents of children with Visual or visual-and-intellectual disabilities). Parent-child dyads will be randomized into two groups: 50 dyads will receive VIPP-V in combination with care-as-usual and 50 dyads will receive care-as-usual. Families with a child (1–5 years of age) with a visual or visual-and-intellectual disability will be recruited for participation in the study. Primary outcome measures are parental sensitivity and the quality of parent-child interaction. Secondary outcome measures are parental self-efficacy, and parenting stress. To assess feasibility of implementation of the intervention the experiences of early intervention workers with regard to using VIPP-V are assessed. Moderator variables are the child’s developmental age, working alliance between parent and VIPP-V intervention worker and empathy of the VIPP-V intervention worker. Data will be collected approximately one week before the intervention starts (T1), one week (T2) and three months (T3) after the intervention. Parent-child dyads in the care-as-usual-only condition will be assessed at the same time points. Both intention-to-treat and completer analyses will be performed.

**Discussion:**

Descriptive findings in pilot cases suggest benefits from VIPP-V, and compatibility with existing services for parents of children with a visual or visual-and-intellectual disability. The current study will provide insight into the effectiveness of this intervention for parents of children with a visual or visual-and-intellectual disability, and, if the intervention is effective, prepare the field for broad-scale implementation.

**Trial registration:**

Nederlands Trial Register NTR4306 (registered 5 December 2013).

## Background

In general, for parents of children with a visual disability, mutual communication is more difficult than for parents with sighted children [1]. The attempts of children with visual disabilities to communicate with their parents are different and sometimes difficult to understand [2]. For example, parents may experience a feeling of rejection because of the lack of eye-contact, the absence of reciprocal smiling or the absence of gaze following as the parent moves around the room. Also, parents may experience their child to be unresponsive to their bids of attention because of the absence of facial expressions and emotional reactions [3]. In Western European countries about 50 % of children with visual disabilities also have an intellectual disability [4, 5]. For parents of children with a visual disability as well as an intellectual disability, interpretation of communication signals in their child may be even more difficult, because of the relatively slow speed at which the child processes (social) information and the delay in or absence of a reaction [1, 6]. These difficulties in understanding and interpreting their child’s behavior and interaction may cause parents to experience increased parenting stress, in turn reducing parental emotional availability, making parents less sensitive and responsive towards their child [1].

Diagnoses of the child’s visual or visual-and-intellectual disability may often come after a period of worry and uncertainty, because of the aberrant behavioral repertoire these children exhibit towards their parents [1]. Nevertheless, the actual diagnosis may often come as a shock, requiring parents to come to terms with possible feelings of sadness, disappointment and guilt [7]. This period around the diagnosis and the associated emotional upheaval coincides with an important period in infants’ lives; a period in which establishing close relationships with caregivers is an important, first developmental issue [8]. A secure attachment relationship provides an optimal basis for adaptive, resilient development [9], which may be particularly relevant for children with disabilities [10, 11].

Despite the fact that several studies have revealed difficulties for parents rearing their child with a visual or visual-and-intellectual disability [1, 3, 6], unfortunately so far little systematic attention has been given to the development of early intervention programs in which parents can learn to relate to their child with a disability in an appropriate and attuned way [2, 12]. Scientific insights on the importance of sensitive parenting and secure parent-child attachments for a positive social-emotional development have accumulated into a solid knowledge base for intervention, both for children with [13] and without disabilities [14]. For children without disabilities this has led to considerable progress in evidence-based intervention programs. A meta-analysis showed that particularly interventions focused on sensitive parenting were effective, even more so than interventions with a broad focus [15]. In addition, this meta-analysis showed that short-term interventions were more effective than long-term interventions (with more than 16 sessions). Based on these findings, Video-feedback Intervention to promote Positive Parenting has been developed (VIPP; [14]). VIPP is an evidence-based attachment-oriented intervention aimed to enhance parental sensitivity, by use of providing personal video-feedback on sensitive responsiveness [14]. This intervention has been adapted for and tested on effectiveness in several subpopulations: for example, parents of children with challenging behavior (VIPP-SD) [16], parents of children with autism (VIPP-AUTI) [17] and parents with a learning disability (VIPP-LD) [18].

In order to meet the needs of parents for support in improving the quality of the relationship with their young child with a visual or visual-and-intellectual disability, the existing Video-feedback Intervention to promote Positive Parenting (VIPP; [14]) has been adapted to Video-feedback Intervention to promote Positive Parenting in parents of children with Visual or visual-and-intellectual disabilities (VIPP-V). The original VIPP and elements of variations on VIPP for several subpopulations were used to tailor this new intervention to the specific needs of families with a young child with a visual or visual-and-intellectual disability. In VIPP-V parents are supported to show more sensitive responsive behavior towards their child by helping them to notice child signals, interpret them correctly, and respond to these signals promptly and appropriately [19]. Showing parents video-recordings of their own interaction with their child has been shown to enhance their insight in the specific needs of their child and improve parental responses [20]. In addition, parents are provided information on the effect of the visual disability on the behavior of their child.

### Trial objective

In the present study we will examine the effectiveness of an attachment-based video-feedback parenting intervention for parents of children with a visual or visual-and-intellectual disability (VIPP-V) by use of a randomized controlled trial (RCT) with two groups: one group will receive VIPP-V in combination with care-as-usual and one group will receive care-as-usual only. Effectiveness is measured in terms of improved parental sensitivity, parent-child interaction quality, parental self-efficacy and decreased parenting stress. Potential moderating variables will be investigated in order to identify predictors of effectiveness. A secondary goal of the study is to assess the feasibility of implementing VIPP-V in regular care and prepare the field for broad-scale implementation if the new intervention is effective.

### Hypotheses

The main hypotheses to be tested are:

- Participation in VIPP-V will be associated with stronger improvement in parental sensitive responsiveness and quality of parent-child interaction than receiving care-as-usual only.
- Parents participating in VIPP-V will show a stronger increase in parental self-efficacy and a stronger decrease in parenting stress than parents receiving care-as-usual only.

## Methods

### Study design

This study is a multicenter RCT with pretest, post-test assessments and follow-up after three months (three time points), including two groups: an experimental group who will receive VIPP-V in combination with care-as-usual, and a control group who will only receive care-as-usual. Randomization will be performed as stratified block randomization with a 1:1 allocation. This trial will be carried out in two national organizations specialized in care for people with visual disabilities and their families (Royal Dutch Visio and Bartiméus). These organizations have offered services to people with visual disabilities and their families since the 1980s and have multiple rehabilitation centers all over The Netherlands. Together, Royal Dutch Visio and Bartiméus provide care for the majority of families eligible for participation in the study (with the exception of families living in the three northern-most counties because of organizational issues).

### Study population

One hundred families with a child (aged 1–5 years) with a visual or visual-and-intellectual disability will be recruited from the population of families receiving early intervention services from Royal Dutch Visio and Bartiméus. For about a quarter of all families receiving care from these organizations, it is very likely that the child also has an intellectual disability. These children will also be included in the study, because at this young age it is often difficult to distinguish an intellectual disability from cognitive slow development and the expectation is that parental sensitivity is equally important for children with and without an intellectual disability.

### Inclusion and exclusion criteria

Families with a child with a visual or visual-and-intellectual disability, aged between 1 and 5 years, who are willing to take part in a video-feedback intervention are eligible for participation in the study. All included children will have a visual disability, as defined according to the World Health Organization (WHO) standards [21]. For children with a learning disability, a clinical psychologist must assess this. Written consent for participation in the study needs to be given by either a parent or, if both parents have custody over the child, by both parents. Parents with visual or auditory disabilities themselves can also participate in the study as extra case studies (in addition to the 100 families that are planned to be included), as it is not yet known how these parents can benefit from the use of video-feedback. Excluded from participation in the study are: families with a child with a developmental age below nine months, because the attachment relationship may not be stable before that age; families with a child who does not live at home: for example due to hospitalization for serious medical problems; parents with an intellectual disability; and multiple children from the same family.

### Interventions

#### Video-feedback Intervention to promote Positive Parenting in parents of children with Visual or visual-and-intellectual disabilities (VIPP-V)

In a collaborative project together with Royal Dutch Visio and Bartiméus, the intervention program Video-feedback Intervention to promote Positive Parenting in parents of children with Visual or visual-and-intellectual disabilities (VIPP-V), was developed. This intervention program was based on the original VIPP [14] and elements of VIPP-SD [16] and VIPP-AUTI [17] were used to make this new intervention applicable for use with families with a young child with a visual or visual-and-intellectual disability. Adaptations were made based on knowledge of the behavior patterns of young children with visual or visual-and-intellectual disabilities, together with clinical experience and knowledge of attachment-based interventions. VIPP-V consists of seven 1.5 hour-sessions. The first five sessions are spent on the original VIPP themes (Attachment and exploration; “Speaking for the child”; Chain of sensitivity; and Sharing of emotions) with an added component related to visual or visual-and-intellectual disabilities each session. The sixth and seventh sessions are booster sessions. The added themes for parents of children with visual or visual-and-intellectual disabilities are: 1) predictability and safety; 2) independence, making demands of the child, and dealing with change and frustration; 3) sharing of attention and joint attention; 4) recognizing and naming emotions and empathy and induction. These added themes focus on skills which (parents of) children with visual or visual-and-intellectual disabilities often have difficulties with. An overview of the themes of the different home-visits can be found in Table 1. The intervention focuses on the primary caregiving parent. The five regular home-visits are scheduled every 2–3 weeks, and the two booster sessions are scheduled in the next two months (every 4–5 weeks). In these booster sessions the other parent is also invited to participate.

**Table 1:** Description of themes in each home-visit in VIPP-V, including additions to the original VIPP

	Themes of VIPP	Additions in VIPP-V
Home-visit 1	Only used for video-taping parent-child interaction. Discussion of interaction starts at home-visit 2	No additions
Home-visit 2	*Exploration	*Predictability
	*Proximity-seeking	*Safety
Home-visit 3	“Speaking for the child”	*Independence
		*Making demands of the child
		*Dealing with change and frustrations
Home-visit 4	*Sensitive responsiveness to child’s signals	*Sharing of attention
	*Chain of sensitivity	*Joint attention
Home-visit 5	*Sharing of emotions	*Recognizing and naming emotions
	*Chain of sensitivity	*Empathy and induction
	*Corrective messages	
Home-visits 6 and 7 (booster sessions)	Repetition	Repetition

For every home-visit VIPP-V intervention workers will use a protocol in which the goals and activities of the home-visit are described. By use of a protocol the intervention is standardized, but the protocol leaves enough flexibility to tailor the intervention to the characteristics and needs of each specific parent-child dyad. Every home-visit starts with video-taping the participating parent and child engaging in several tasks (for example playing together, teaching the child a task). After every home-visit the VIPP-V intervention worker prepares feedback on the video-taped interactions. This feedback is discussed in the next home-visit, directly after video-taping new material for the home-visit thereafter. Besides feedback the intervention worker also provides information and tips regarding sensitive responsiveness and visual or visual-and-intellectual disabilities. At the end of the intervention, parents receive a brochure with a summary of the most important elements discussed in intervention, including several tips, and a USB-stick with the video-recordings.

Leiden University and VU University Amsterdam have extensive experience in training professionals in VIPP and maintaining treatment fidelity through regular supervision, both within and between universities. Researchers of Leiden University have trained eight special education and behavioral experts from Royal Dutch Visio and Bartiméus as coaches in VIPP in a five-day training. During this training period all eight coaches practiced their newly acquired skills in a pilot family, and received five intervision sessions of 3 hours each with fellow coaches and three supervision session of 3.5 hours each. At the end of the training period trainers from Leiden University checked and approved the video-recordings and scripts of one of the seven home-visits of all coaches. Then, two of these coaches (one of each organization), in alternating collaboration with the second author, have trained fifteen early intervention workers in conducting VIPP-V. Early intervention workers received five days of training in VIPP-V over a period of two months. During this same period the intervention workers completed a VIPP-V intervention with families who, due to age, could not be included as participants in the study. Each of the five training days was dedicated to one home-visit (the last two of the seven sessions are booster sessions in which previous topics are repeated), and during the training all intervention workers participated in intervision sessions on each VIPP-V home-visit. Two of the trainers provided feedback on one of the scripts and on a video-recording of the feedback moment with the parent of each intervention worker. The VIPP-V training materials, video-recordings, scripts and provided feedback of the trainers of all intervention workers were finally sent to researchers of Leiden University for a check, and approved. These 23 VIPP-V intervention workers will provide VIPP-V to the different participating families. In addition, the eight VIPP-V coaches will participate in three supervision meetings with the fifteen early intervention workers during each VIPP-V trajectory.

#### Care-as-usual

Families randomized into the experimental condition will receive both VIPP-V and care-as-usual, while families randomized into the control condition will receive only care-as-usual. At Royal Dutch Visio and Bartiméus families receive care for a wide range of issues and topics, such as mobility training and magnifier training for the child, and guidance for parents on parenting issues, play behavior and choice of schools. At pretest, post-test and follow-up all families are asked about the kind and amount of care they have received recently. An analysis and comparison will be made of the received care-as-usual in both conditions. If necessary, kind and amount of care-as-usual will be controlled for in analyses.

### Measures

Primary outcome measures, secondary outcome measures, outcomes regarding feasibility of implementation, and measures of moderating variables and control variables can be distinguished in this study. The instruments include several questionnaires for parents and one observation measure for parent and child at each assessment, several questionnaires for the VIPP-V intervention worker at pretest and post-test and one questionnaire for the early intervention worker who provides care-as-usual in the family at pretest.

After the start of the trial changes were made in the designation of primary and secondary outcome measures. To increase feasibility of the study, in the early stages of the trial the original proposal was changed so that parental sensitivity and quality of parent-child interaction is now coded with all National Institute of Child Health and Human Development Scales (NICHD-scales), and parental self-efficacy and parenting stress are secondary outcome measures rather than primary outcome measures.

### Primary outcome measures

The main research question of this study is whether parents of a child with a visual or visual-and-intellectual disability will benefit from participating in VIPP-V in terms of higher parental sensitivity and better parent-child interaction quality compared to receiving care-as-usual only.

#### Parental sensitivity and quality of parent-child interaction

At pretest, post-test and follow-up the Three Boxes-procedure [22, 23] will be used to observe parental sensitivity and quality of parent-child interaction. Since all assessments take place during home-visits instead of in the lab, the originally used boxes in the Three Boxes-procedure were changed for bags in this study for ease of transportation. For this procedure parent-child dyads are offered three bags with toys. Small adaptations have been made to the originally used toys to make the play sets also appropriate for children with visual disabilities. The first bag contains a tactile reading book (different books are used for younger children (1–2 years) and older children (3–4 years)); a play tea set is in the second bag (this set consists of tea cups of different materials: for example a metal tea cup, a wooden tea cup and a plastic tea cup, so blind children will also have enough possibilities for exploration; the tea set for older children consists of more items); a set of Duplo building blocks is in the third bag (more blocks are in the play set for older children). Parents are instructed to play with their child with the toys in the three bags in a specified order (each bag is numbered 1–3). The **National Institute of Child Health and Human Development Scales (NICHD-scales)** will be used to code parental sensitivity and quality of parent-child interaction in the Three Boxes-procedure [24]. These scales will be scored on a 7-point Likert-scale, ranging from *“very low (1)”* to “*very high (7)”* Coders will be trained by the fourth author (Prof. Dr. Schuengel), who has established reliability in previous studies [25, 26]. Tapes will be randomly assigned to coders, and coders will be blind to condition and assessment. In a previous study Cronbach’s alpha for a composite parental sensitivity measure (sum of scales) ranged from 0.70 to 0.78, and high intercoder reliability was achieved, ranging from 0.83 to 0.87 [22].

### Secondary outcomes

Secondary outcome measures focus on whether parents of a child with a visual or visual-and-intellectual disability will benefit from participating in VIPP-V in terms of higher parental self-efficacy and lower parenting stress compared to receiving care-as-usual only.

#### Parental self-efficacy

The **Self-efficacy in the Nurturing Role questionnaire (SENR)** [27] will be administered to parents at pretest, post-test and follow-up to measure parental self-efficacy. This questionnaire consists of 16 items, which can be rated on a 7-point scale ranging from *“not at all applicable to me (1)”* to *“totally applicable to me (7).”* Items capture parents’ perceptions of their competence on basic skills required in taking care of their child. Items were modified slightly to be appropriate for parents parenting children aged 1–5 years, instead of infants. Examples of items are: *“I feel competent in my role as a parent”* and *“Touching, holding and being affectionate with my child is comfortable and pleasurable for me.”* In previous research with mothers of infants internal reliability was high, with a Cronbach’s alpha ranging from 0.86 to 0.89 [28].

#### Parenting stress

At pretest, post-test and follow-up parents will be asked to fill out the shortened Dutch version of the **Parenting Stress Index (PSI)** [29, 30] to measure the overall stress experienced in parenting. This questionnaire consists of 25 items divided in a scale for child-related parenting stress and a scale for parent-related parenting stress. An example of an item regarding child-related parenting stress is: *“My child cries or fusses more often than other children*,*”* and an example of an item regarding parent-related parenting stress is: *“I often feel that I cannot handle things.”* Parents are asked to answer on a 6-point scale, ranging from *“totally disagree (1)”* to *“totally agree (6).”* Internal consistency in previous research ranged from 0.92 to 0.95 and validity seems acceptable [30].

### Outcomes regarding feasibility of implementation

If study-results show VIPP-V to be an effective intervention a second goal of this study is to assess the feasibility of implementing VIPP-V in the regular care provided by early intervention workers at Royal Dutch Visio and Bartiméus.

#### Experiences of early intervention workers with VIPP-V during early intervention

An adaptation of the **Social Validity Scale** [31, 32] will be administered at post-test to VIPP-V intervention workers to assess the desirability, applicability, clarity, efficiency and burden of integrating VIPP-V in the current range of care provided by Royal Dutch Visio and Bartiméus. This questionnaire consists of 35 questions and 18 statements. The questions cover four topics (evaluation of the ideas behind the VIPP-V intervention, evaluation of offering VIPP-V in the participating family, subjective evaluation of the effectiveness of VIPP-V in the participating family and evaluation of completing the different questionnaires) and can be answered on a 5-point scale. The statements are formulated more generally and focus on three topics (application of acquired VIPP-V skills, subjective evaluation of the effectiveness of VIPP-V, usefulness of VIPP-V) and can be answered on a 3-point scale.

### Measures of moderating variables

Data will be gathered regarding demographic variables, child’s developmental age, working alliance between parent and VIPP-V intervention worker and empathy of the VIPP-V intervention worker to determine whether there are certain subgroups for whom or circumstances under which VIPP-V is most effective.

#### Demographic variables

Before pretest, parents complete a 14-item self-designed demographic questionnaire. Questions are included regarding age and gender of the parent, child and other family members, cultural and socio-economic background, health of parents, nature and severity of the disability of the child, medical background of the child, use of medication of the child and possible disabilities of other family members. This questionnaire is administered before pretest, because parents who have visual or auditory disabilities themselves will be included as extra case studies, children with a severe intellectual disability (developmental age below nine months) will be excluded from participation and randomization will be done stratified on the chronological age of the child (to control for the number of years the parent has had experience in interacting with the child).

#### Child’s developmental age

For parents it will sometimes be difficult to assess the developmental age of their child correctly on the demographic variables questionnaire. Therefore, the early intervention worker who provides care-as-usual in the family will be asked to fill out the Dutch screening version of the **Vineland Adaptive Behavior Scales (VABS)** [33, 34] around pretest to obtain a more objective measure for children’s developmental age. If an early intervention worker is not available, a professional who is otherwise closely involved with the child, such as a teacher, will be asked to fill out this questionnaire. This screening instrument consists of 93 items to measure children’s adaptive behavior on four domains: Communication, Daily Living Skills, Socialization and Motor Skills. A previous study on the psychometric properties of this Dutch translation administered to parents showed good validity, excellent internal consistency (Cronbach’s alpha = 0.99) and test-retest reliability (Intraclass Correlation Coefficient (ICC) = 0.95) [35].

#### Working alliance between parent and VIPP-V intervention worker

The therapeutic alliance has been found to predict treatment outcomes and seems an important aspect of successful intervention [36]. Therefore, the Dutch translation of the short version of the **Working Alliance Inventory (WAI)** [37–40] will be administered to both the parent and the VIPP-V intervention worker at post-test to assess their feelings of working alliance. This questionnaire consists of 12 items, measuring general alliance as well as three specific aspects of alliance (Bond, Goals and Tasks). An examples of an item is: *“We* (the parent and VIPP-V intervention worker) *have established a good understanding of the kind of changes that would be good for me and my child.”* Items are scores on a 5-point scale, ranging from *“never (1)”* to *“always (5).”* Validity and reliability of the original English version has been demonstrated to be good [37]. During VIPP-V the VIPP-V intervention worker also fills out a notebook and profile of the participating parent after every home-visit to report on the course of the visit, the collaboration with the parent and the progress the parent has made between visits. This notebook and profile will be used to support the data gathered on the WAI.

#### Empathy of VIPP-V intervention worker

Empathy of the VIPP-V intervention worker can be expected to be associated with the relationship between the VIPP-V intervention worker and the parent [41] and may therefore, contribute to the effectiveness of intervention. To assess empathy of the VIPP-V intervention workers the short version of the **Empathy Quotient (EQ-short)** [42, 43] is used at pretest and post-test. This questionnaire consists of 22 items which measure how easily one picks up other people’s feelings and how strongly one is affected by those feelings. Items are scored on a 4-point scale, ranging from *“totally disagree (1)”* to *“totally agree (4).”* An example of an item is: *“I can easily tell if someone else wants to enter a conversation.”* The short version of the EQ-short has been shown to have good validity and reliability (Cronbach’s alpha = 0.90) [43].

### Measures of control variables

Potential stressful experiences during intervention and treatment integrity will be measured and used as control variables.

#### Potential stressful experiences

To control for the effect of stressful experiences in the child’s life on the effectiveness of the VIPP-V intervention, the VIPP-V intervention worker is asked to check any potential stressful experiences in the child’s life on a checklist at pretest and post-test.

#### Treatment integrity

All home-visits are video-taped to provide video-feedback. These video-tapes will also be used to assess whether the intervention is carried out according to the manual. Ten percent of the video-tapes will be randomly selected to code treatment integrity.

### Procedure

To recruit families for participation in the study Royal Dutch Visio and Bartiméus will send an information package, including a letter, brochure and consent form developed by the researchers, as well as a cover letter from the organization to all families in their databases who meet the inclusion criteria. In addition to this broad approach, also all early intervention workers are asked to personally inform the families in their caseload who are eligible for participation about the study. If families decide to participate in the study, they sign a consent form and fill out a demographic questionnaire. When they return these forms to the university, their personal data becomes known to the researchers. Families who are approached for participation, but do not wish to participate in the study, remain anonymous to the researchers. If parents have questions regarding the study they can phone or email one of the eight VIPP-V coaches working at Royal Dutch Visio and Bartiméus or one of the researchers.

**Fig. 1:**
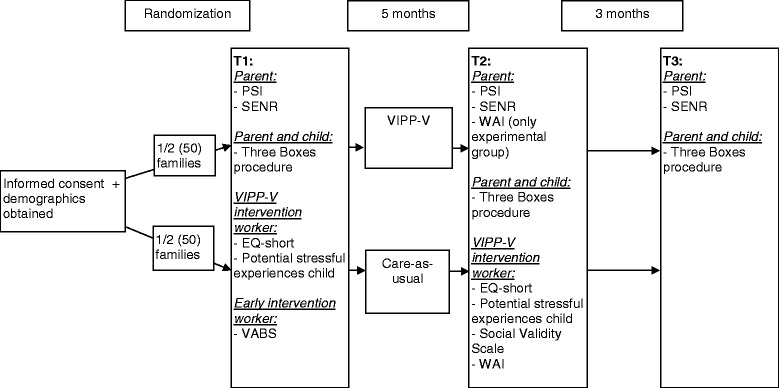
Research procedure. EQ-short = Empathy Quotient short questionnaire; PSI = Parenting Stress Index; SENR = Self-efficacy in the Nurturing Role questionnaire; VABS = Vineland Adaptive Behavior Scales; VIPP-V = Video-feedback Intervention to promote Positive Parenting in parents of children with Visual or visual-and-intellectual disabilities; WAI = Working Alliance Inventory

After informed consent and demographics are obtained, families are enrolled in a block of 16–18 families. In both organizations three blocks of 16–18 families will be formed, to include a total of 100 families in the study. When the maximum number of families for the block is reached, families will be randomized, and a few weeks later the intervention will start. The intervention will start on three different occasions over the year, so each intervention worker provides VIPP-V to approximately one family at a time. After randomization families are informed in which condition they will participate through a letter by the research team. A research assistant makes an appointment with the family for the pretest assessment (T1). After the pretest the intervention starts. After about five months the intervention is finished and a post-test takes place. Three months later an appointment is made for the follow-up assessment. Families participating in the control condition will be assessed at the same time points (see Fig. 1).

Assessments take place in the home of the family and consist of a computerized administration of questionnaires for the parent, which takes about 20–30 minutes, and a parent-child interaction play task of about 15 minutes (see Measures for a more extensive description of the study instruments). After each assessment parents receive a small gift (for example a puzzle, stuffed animal or book) for their child. During the study, participating families will be informed about the study progress through a newsletter. At the end of the study, all families will receive a general report on the effect of VIPP-V. This procedure was approved by the Medical Ethics Committee (METc VUmc 2013/449/NL47334.029.13).

### Randomization

Randomization will be done by use of a computerized random number generator by the first author. Using stratified randomization, equal randomization of families from both Royal Dutch Visio and Bartiméus will be achieved, as well as equal representation of children with varying chronological ages. Stratified randomization reduces the risk for baseline imbalance between conditions [44]. For each organization separately, the random allocation list will be generated in three blocks with a 1:1 allocation, based on three periods in which the intervention will be offered. After the second block a check will be done for baseline imbalances between conditions, so, if necessary, these imbalances can be corrected in the final block. Each block will be randomized roughly three weeks before the start of the intervention. The numbers on the list will be paired with the participating families in order of the date on which informed consent is obtained.

### Blinding

Parents agree to participate in the study before randomization and without knowing in which condition they will participate. The condition in which parents will participate will be disclosed to them directly after randomization of the block they belong to (roughly three weeks before the start of the intervention). Every ID-number assigned to families is independent of condition. Researchers coding and analyzing the observation data will be blind to the condition of parents and children, as well as the assessment (pretest, post-test, follow-up). Research assistants conducting the assessments are not blinded to the condition or assessment, because different questionnaires are used for families participating in the intervention condition and in the control condition (families in the control condition are not asked about working alliance of the VIPP-V intervention worker) and per assessment. Intervention workers are, necessarily, not blind to randomization allocation.

### Sample size calculation

The total sample size is based on an expected effect size of *d* = 0.33 (*f* = 0.17), based on a meta-analysis on RCTs of interventions focusing on improving maternal sensitivity [15]. Based on an alpha of 0.05 and a sample of 100 families, adequate statistical power will be achieved (0.97) for testing the significance of an interaction effect between the between-subject factor (experimental versus control condition) and the within-subject factor (assessment: pretest, post-test, follow-up) in repeated measures analysis of variance (ANOVA) using the full sample of 100 families. If, due to circumstances such as drop-out, illness of VIPP-V intervention workers or unexpected organizational aspects, the number of families drops, with an effect size this high, high power (0.80) can still be achieved with 58 families for the main research question regarding effectiveness of intervention on parental sensitivity. However, to also perform moderator analyses with adequate power we aim to include 100 families in the study.

### Drop-outs

All families who consented to participate in the study will be followed from pretest to the follow-up assessment eight months later. If families drop out of the intervention, a post-test and follow-up assessment will still be planned if possible. If, for some reason, the assessments cannot take place the principle of last value carried forward will be applied. The reasons for drop-out will be documented and analyzed and the involved organization will be notified of these reasons in order to use this information to possibly improve the implementation of the intervention. Intention-to-treat analyses will be conducted to account for drop-out.

### Data analysis

All analyses will be done using the software program SPSS (version 21.0). Baseline characteristics will be described in descriptive analyses. Before analyzing, differences in baseline characteristics between the experimental and control condition will be checked. If differences are found, they will be reported and controlled for in further analyses. Before analyzing, outliers will be checked and winsorized if necessary. For analyses of the primary and secondary outcome measures of the study (parental sensitivity, parent-child interaction quality, parental self-efficacy and parenting stress) ANOVA repeated measures or multilevel analyses will be used, depending on the level of clustering in the data. Intention-to-treat and completer analyses will be performed. The primary and secondary outcome measures of families in the experimental condition will be compared with their own scores on a previous assessment, as well as with scores of families in the control condition at the same assessment. Change over time from pretest to post-test and from pretest to follow-up in parental sensitivity, quality of parent-child interaction, parental self-efficacy and parenting stress will be assessed in the experimental group, and compared with the control group. Results will be described as rates of change. The outcome measure regarding feasibility of the intervention ‘Experiences of VIPP-V intervention workers with VIPP-V during early intervention’ will be reported in descriptive analyses. Multivariate regression analysis or multilevel moderator analyses will be performed with different predictors (demographic variables, child’s developmental age, working alliance between parent and VIPP-V intervention worker, empathy of the VIPP-V intervention worker) to study for whom and under which circumstances VIPP-V intervention is most effective. For these analyses only the data of the families in the VIPP-V intervention condition will be used.

### Data management and monitoring

All data will be collected through computerized assessments, which obviates the need for double data entry. A Data Monitoring Committee has not been established, because of minimal risks for involved participants. The funding organization of this study (ZonMw InZicht) will conduct a site visit halfway through the project to check on its progress. This study is embedded in the EMGO+ Institute for Health and Care Research of the VU University Medical Center in Amsterdam, The Netherlands. The quality committee of EMGO+ has created an electronic quality assurance handbook to uniform the conduct and safeguard the quality of research within the institute. In addition, EMGO+ does random project audits to assure the quality of all projects. This study adheres to the quality handbook and anticipates the possibility of being randomly audited.

### Protection of data privacy

After informed consent for participation in the study is obtained, all participating families will be assigned a number. Key lists of number – personal data combinations will be stored separately from the data and will be deleted when all data analyses are completed. Data will be analyzed in a way that no conclusions can be drawn about individual participants. All data will be stored on a password-protected server, and if applicable, in lockable cabinets in lockable rooms. All (assistant) researchers with access to the data will sign a non-disclosure statement, which states they will not disclose any information about research participants to a third party.

### Publication policy

Results of this study will be published in peer-reviewed, international journals. To bring the study results to the attention of practitioners who need to implement them, we also plan to publish at least one paper in a national journal within the field of visual disabilities/intellectual disabilities studies. Results will be presented at international scientific conferences and national conferences within the field of disability research. All authors will have access to the full dataset and will have equal opportunity to publish on the dataset. The use of professional writers is not planned.

### Ethical considerations

The Medical Ethics Committee of the VU University Medical Center in Amsterdam, The Netherlands has approved the study protocol (METc VUmc 2013/449/NL47334.029.13). Possible future changes to the study procedures will be proposed to the Medical Ethics Committee as amendments, and will be described and discussed in the publication of the study results hereinafter.

## Discussion

As the need for support in families with a child with a visual disability is clear [1–3], these families should receive the best possible support. Although several early intervention services have been developed that offer support in adapting to the disability and promoting optimal development, these interventions are not yet evidence-based and not transferable through written protocols. This study aims to provide insight into the effectiveness of an adaptation of Video-feedback Intervention to promote Positive Parenting (VIPP) for this specific population (VIPP-V) and to fill a gap in the knowledge about evidence-based treatment for families with a young child with a visual or visual-and-intellectual disability.

VIPP-V is a short intervention, with only seven home-visits over a period of five months. This intervention can be implemented on top of care-as-usual. Short-term interaction-based interventions (fewer than 16 sessions) have been shown to be effective in increasing parental sensitivity and children’s attachment security [15]. A short-term intervention limits the intervention burden for participating families.

Before implementation of this new intervention, a small survey was carried out among 16 parents with a child aged 4–5 years, who already received care from Royal Dutch Visio or Bartiméus. In this survey VIPP-V was described and presented as an intervention which may be offered in the future. Parents were asked how useful they rated this intervention and how likely they would participate. Ratings showed VIPP-V as highly useful (a mean score of 8 out of 10) and parents responded they would likely participate in the intervention if it would be offered (a mean score of 6 out of 10). This small survey supports the relevance of efforts to develop interventions aimed at improving parent-child interaction in families with a child with a visual or visual-and-intellectual disability. Next, a pilot-study of this new intervention was conducted among parents (*N* = 10) and VIPP-V intervention workers (*N* = 8). Participating parents and VIPP-V intervention workers reported positive results after participating in VIPP-V; both parents and intervention workers rated the individual approach with video-feedback and the provision of information on the visual disability as (very) effective (a mean score of 4 out of 5). In addition, parents and VIPP-V intervention workers indicated that the attitude of the parent towards the child, as well as the parent-child interaction was improved. Also, both parents and VIPP-V intervention workers reported an increase in parental self-efficacy. These descriptive findings of both the survey and the pilot suggest benefits from VIPP-V, compatibility with existing services for parents of children with a visual or visual-and-intellectual disability and provide a basis for further research on the effectiveness of this video-feedback intervention.

A strength of the study is the close collaboration with two national organizations, both with multiple rehabilitation centers throughout The Netherlands. In case our results show the effectiveness of VIPP-V, this new intervention can be immediately continued as a component of already offered services for parents of a child with a visual or visual-and-intellectual disability. National centers providing support for parents of children with a visual disability have the responsibility to provide evidence-based interventions during early intervention. Close collaboration between practice and science is, therefore, key to respond to the wishes of parents for more information and help in improving parent-child interaction quality.

In conclusion, this study will provide insight into the effectiveness of an attachment-based video-feedback intervention for parents of children with a visual or visual-and-intellectual disability and, if the intervention is effective, prepare the field for broad-scale implementation.

## Status of the trial

The study started in September 2013. After being granted permission by the Medical Ethics Committee to start with the inclusion of research participants, the first families were included in February 2014. Currently, data collection is in progress. The main results are expected to be published in the beginning of 2016.

## Abbreviations

ANOVA: Analysis of variance
EQ-short: Empathy Quotient questionnaire short-version
ICC: Intraclass Correlation Coefficient
METc: Medical Ethics Committee
NICHD scales: National Institute of Child Health and Human Development Scales
PSI: Parental Stress Index
RCT: Randomized Controlled Trial
SENR: Self-efficacy in the Nurturing Role questionnaire
T1: First assessment
T2: Second assessment
T3: Third assessment
USB: Universal Serial Bus
VABS: Vineland Adaptive Behavior Scales
VIPP: Video-feedback Intervention to promote Positive Parenting
VIPP-AUTI: Video-feedback Intervention to promote Positive Parenting adapted to Autism
VIPP-LD: Video-feedback Intervention to promote Positive Parenting and sensitive disciplining for parents with Learning Difficulties
VIPP-SD: Video-feedback Intervention to promote Positive Parenting and Sensitive Discipline
VIPP-V: Video-feedback Intervention to promote Positive Parenting in parents of children with Visual or visual-and-intellectual disabilities
VUmc: VU University Medical Center
WAI: Working Alliance Inventory
WHO: World Health Organization
